# Myeloid but not hepatocytic CD38 is a key driver for hepatic ischemia/reperfusion injury

**DOI:** 10.1038/s41392-025-02233-8

**Published:** 2025-05-09

**Authors:** Qi-Hang Zhao, Ya-Ting Zhang, Ke Wen, Qi Ding, Zi-Ying Chen, Dilinuer Tula, Jia-Hui Li, Juan Zhou, Yun-Fei Xiao, Xiao-Hui Guan, Ke-Yu Deng, Ling-Fang Wang, Hong-Bo Xin

**Affiliations:** 1 National Engineering Research Center for Bioengineering Drugs and the Technologies, Institute of Translational Medicine, Jiangxi Medical College, Nanchang University, Nanchang 330031, China; 2 School of Life Science, Nanchang University, Nanchang 330031, China; 3 School of Pharmacy, Jiangxi Medical College, Nanchang University, Nanchang 330031, China; 4 Department of Gynecology and Obstetrics, The First Affiliated Hospital, Jiangxi Medical College, Nanchang University, Nanchang 330006, China

**Keywords:** Gastrointestinal diseases, Cell biology

## Abstract

Hepatic ischemia-reperfusion injury (HIRI) is a critical condition that often occurs during liver transplantation and surgical liver resection. However, its mechanism has not been fully elucidated. Nicotinamide adenine dinucleotide (NAD^+^), functioning as a coenzyme or cofactor, is crucial for both redox and non-redox processes. In mammals, CD38 serves as the primary enzyme responsible for NAD^+^ degradation. In this study, we reported that the absence of CD38 markedly reduces HIRI in CD38 global knockout (CD38^KO^) and CD38 myeloid-specific knockout (CD38^MKO^) mice, but not in CD38 hepatocyte-specific knockout (CD38^LKO^) mice compared with the control (CD38^fl/fl^) mice by suppressing HIRI-induced hepatic oxidative stress, inflammatory responses, and pyroptosis. The findings were corroborated by a noticeable decrease in levels of alanine aminotransferase (ALT), aspartate transaminase (AST), and lactate dehydrogenase (LDH), along with reduced necrosis. Besides, we found that the expressions of SIRT1 and its downstream targets, p53 and PPARγ, were elevated in the liver tissues of CD38^KO^ and CD38^MKO^ mice compared to CD38^fl/fl^ mice, while the acetylation levels of p53 were reduced. Furthermore, we demonstrated that myeloid CD38 deficiency not only promoted M2-type polarization and inhibited M1-type polarization of macrophages but also suppressed NLRP3-mediated pyroptosis by triggering NAD^+^/SIRT1 signaling in macrophages, resulting in the reduction of oxidative stress, inflammation, and pyroptosis in the liver, ultimately protecting against HIRI. This study highlights myeloid CD38 as a promising target for the prevention and treatment of HIRI clinically.

## Introduction

Liver transplantation and hepatectomy are the best options for the treatment of patients who suffer from various forms of end-stage liver disease as well as malignant liver tumors.^1^ Although advancements in immunosuppression and medical care have increased patient survival rates to approximately 75% for 5 years, HIRI, an innate immune-mediated response occurring during transplantation, continues to be a risk factor affecting clinical outcomes and exacerbating the shortage of life-saving transplantable organs.^2^ HIRI occurs when the perfusion of blood flow is restored after a certain period of hypoxia stress.^3^ Firstly, due to the lack of oxygen supply, cells experience enhanced anaerobic respiration and mitochondrial dysfunction, which in turn leads to the imbalance of ion homeostasis, the dysfunction of the sodium-potassium pump, and the collapse of membrane potential, resulting in calcium overload, and activating calcium-dependent proteases to degrade the protein skeleton, ultimately leading to ATP depletion, degradation of membrane proteins, destruction of cell structure, and DNA damage.^4^ Secondly, the restoration of blood flow brings a large amount of oxygen, which promotes the generation of various reactive oxygen species (ROS), causing peroxidative damage to cell lipids, proteins, nucleic acids, etc., and leading to liver metabolic disorders and triggering an interconnected sterile inflammatory cascade reaction. In addition, damaged hepatocytes in the ischemic injury stage release DNA fragments and various damage-associated molecular patterns (DAMPs), activating the immune response, stimulating the release of inflammatory molecules and the recruitment of immune cells, leading to an inflammatory surge that further exacerbates cellular damage.^5^ Nevertheless, the exact mechanisms have not been fully clarified, and the effective therapeutic strategies or medications for HIRI are still unavailable.^6^

A recent study emphasizes the importance of macrophages in the pathophysiology of HIRI.^7,8^ Targeting macrophage-mediated pyroptosis or polarization has been demonstrated to be a promising therapeutic strategy for mitigating ischemia-reperfusion injury. Classically activated M1 macrophages, driven by TLR4/NF-κB signaling, produce pro-inflammatory mediators that amplify tissue injury during the early phase.^9,10^ Conversely, alternatively activated M2 macrophages can alleviate HIRI injury through the modulation of anti-inflammatory cytokine release, including IL-10 and IL-4.^11^ Dysregulation of this phenotypic switch disrupts the inflammatory balance, prolonging liver injury and impairing functional recovery.^12^ Notably, recent studies have shown that the regulation of macrophage polarization effectively affected ischemia-reperfusion injury, in which HO-1 improved ischemia-reperfusion injury by inhibiting macrophage M1 polarization.^13^ Pharmacological modulation of macrophage polarization, such as supplementation of Liraglutide, also effectively regulated macrophage polarization and improved ischemia-reperfusion injury.^14^ However, the plasticity and the context-dependence of macrophages make its clinical application still remain to be explored and require further investigation.^15^

CD38 is a type II transmembrane glycoprotein composed of a single polypeptide chain with a molecular mass of 45 kDa. About 90% of CD38 is distributed on the cell membrane, and about 10% of CD38 is distributed on other organelles.^16^ CD38 on the cell membrane can be divided into extracellular and intracellular parts, and the main functional domain is in the extracellular region. As an intracellular NAD⁺ hydrolase, CD38 hydrolyzes NAD^+^ to cADPR (Cyclic ADP-ribose) and NAADP (Nicotinic acid adenine dinucleotide phosphate), which participate in regulating various biological functions related to Ca^2+^ signals in cells. We previously reported that knockout or inhibition of CD38 protected hearts from ischemia-reperfusion injury^17^ and high-fat diet-induced oxidative stress^18^ through increasing intracellular NAD^+^ levels in mice. We also observed that the absence of CD38 protected mice from high-fat diet-induced nonalcoholic fatty liver disease.^19^ Besides, CD38 might serve as a potential marker of M1 macrophages due to its regulation of macrophage functions such as proliferation, aggregation, and polarization.^20^ However, the role of CD38 in HIRI has not been explored. The metabolic disorder of NAD⁺, an essential coenzyme involved in cellular redox processes and energy metabolism, has been implicated in the pathogenesis of HIRI. Recently, Yuan’s group observed that NAD⁺ deprivation was associated with ischemic hepatic peroxidation.^21^ Sirtuin 1 (SIRT1), a type III histone deacetylase that used NAD^+^ as a substrate, protected the liver from HIRI by reducing NLRP3-driven inflammation.^22^ In clinical settings, the elevated SIRT1 levels were associated with a reduced proinflammatory response in the post-reperfusion condition, suggesting its role in preserving liver function and improving patient survival.^23^ Therefore, it is important to further explore the function of CD38 in HIRI.

In this study, we utilized global as well as tissue- and cell-specific CD38 knockout mice to explore the functions and mechanisms of CD38 in HIRI. Our results revealed that CD38 expression was upregulated in liver tissues following HIRI. The lack of CD38 in global or myeloid cells, but not in hepatocytes, mitigated HIRI by diminishing oxidative stress, inflammation, and pyroptosis in mice. Furthermore, we demonstrated that myeloid-specific deletion of CD38 is essential for alleviating HIRI by activating the SIRT1-p53/PPARγ signaling pathways. In summary, our results strongly indicate that targeting CD38 on myeloid macrophages holds the potential to offer novel perspectives and avenues for the treatment of HIRI.

## Results

### Global or myeloid but not hepatocytic CD38 deficiency alleviates hepatic ischemia-reperfusion injury in mice

**Fig. 1:**
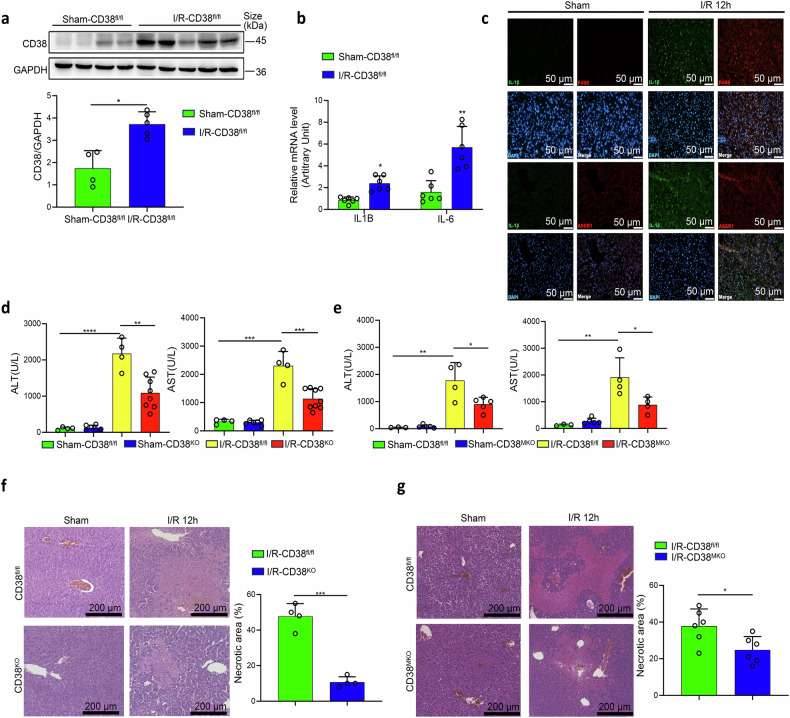
CD38 deficiency alleviated hepatic ischemia-reperfusion injury (HIRI) in mice. **a** CD38 expressions were determined by Western blotting and quantitative analysis in liver tissues after ischemia/reperfusion (I/R) injury. **b** The mRNA expressions of IL1B and IL-6 in liver tissues after I/R injury. **c** Representative fluorescence images of IL-1β/F4/80 and IL-1β/ASGR1 were taken from CD38^KO^ mice subjected to HIRI, respectively. **d**, **e** Serum ALT and AST were measured in CD38^KO^ and CD38^MKO^ mice subjected to HIRI. **f**, **g** Representative H&E staining images and the quantitative analysis of liver ischemic necrosis were taken from CD38^KO^ and CD38^MKO^ mice subjected to HIRI, respectively. Data are shown as means ± SEM, *p < 0.05, **p < 0.01 and ***p < 0.001, *n* = 3–9 per group

To investigate the roles of CD38 in HIRI, a mouse model was generated following a previously established protocol.^24^ The results showed that the protein (Fig. 1a) and mRNA (Supplementary Fig. 1a) expressions of CD38 were increased in liver tissues of the HIRI model mice, along with the increased mRNA expressions of the pro-inflammatory factors IL1B and IL-6 (Fig. 1b). More importantly, the generated IL-1β was predominantly co-localized with macrophages marked by F4/80, rather than co-localized with hepatocytes marked by ASGR1 (Fig. 1c), indicating that the upregulation of CD38, especially in macrophages, might be implicated in the development of HIRI.

Next, global CD38 knockout (CD38^KO^), myeloid-specific CD38 knockout (CD38^MKO^), and hepatocyte-specific CD38 knockout (CD38^LKO^) mice were generated to examine the roles of CD38 in HIRI. The deletion of CD38 in CD38^LKO^ (Supplementary Fig. 1b) and CD38^MKO^ (Supplementary Fig. 1c) was confirmed by western blotting analyses in mice. The results revealed that CD38 deficiency significantly reduced HIRI-induced elevations of serum ALT and AST in CD38^KO^ (Fig. 1d) and CD38^MKO^ (Fig. 1e), but not CD38^LKO^ (Supplementary Fig. 1d) mice compared to CD38^fl/fl^ mice. The similar alterations of LDH were also observed in CD38^KO^ mice (Supplementary Fig. 1e). Additionally, H&E staining demonstrated that CD38 deficiency remarkably inhibited HIRI-induced sinusoidal cell congestion, vacuolization and hepatocyte necrosis of liver tissues in CD38^KO^ (Fig. 1f) and CD38^MKO^ (Fig. 1g), but not CD38^LKO^ (Supplementary Fig. 1f) mice. These results demonstrated that CD38 deficiency improved I/R-induced liver injury in CD38^KO^ and CD38^MKO^, but not CD38^LKO^ mice, suggesting that macrophage CD38 could be an important contributor to HIRI in vivo.

### Global or myeloid CD38 deficiency suppresses IR-triggered hepatic oxidative stress and inflammation

**Fig. 2:**
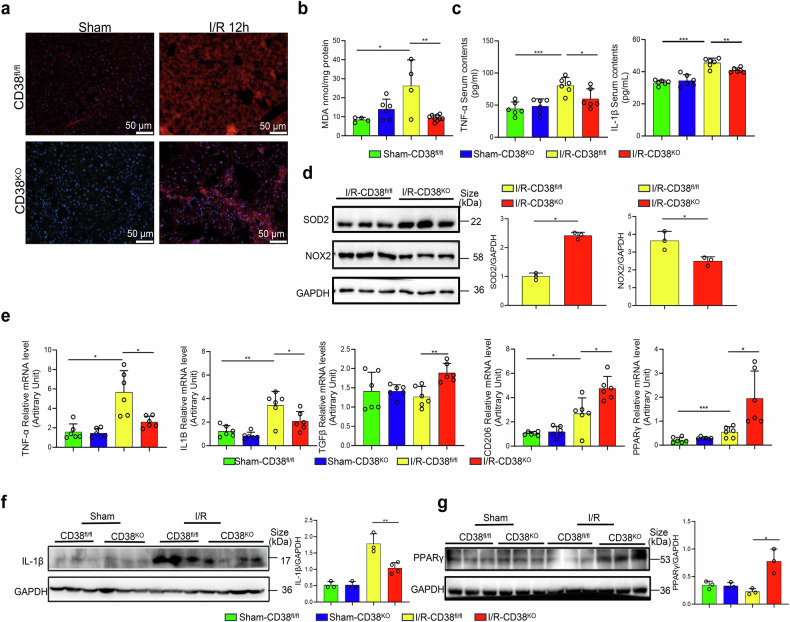
Global CD38 deficiency inhibited oxidative stress and inflammation during HIRI. **a** Representative DHE staining images were taken from CD38^KO^ and CD38^fl/fl^ mice subjected to HIRI, respectively. **b** MDA contents were quantitatively measured in liver tissues from CD38^KO^ and CD38^fl/fl^ mice after I/R injury. **c** Serum TNF-α and IL-1β were detected by Elisa in CD38^KO^ and CD38^fl/fl^ mice after HIRI. **d** The protein expressions and the quantitative analysis of NOX2 and SOD2 were determined by western blot in liver tissues from CD38^KO^ and CD38^fl/fl^ mice after HIRI. **e** The mRNA expressions of TNF-α, IL1B, TGF-β, CD206, and PPARγ were determined by QPCR in CD38^KO^ and CD38^fl/fl^ mice after HIRI, respectively. **f**, **g** The expressions and the quantitative analysis of IL-1β and PPARγ were detected by Western blot in liver tissues in CD38^KO^ and CD38^fl/fl^ mice after HIRI, respectively. Data are shown as means ± SEM, *p < 0.05, **p < 0.01 and ***p < 0.001, *n* = 3–8 per group

**Fig. 3:**
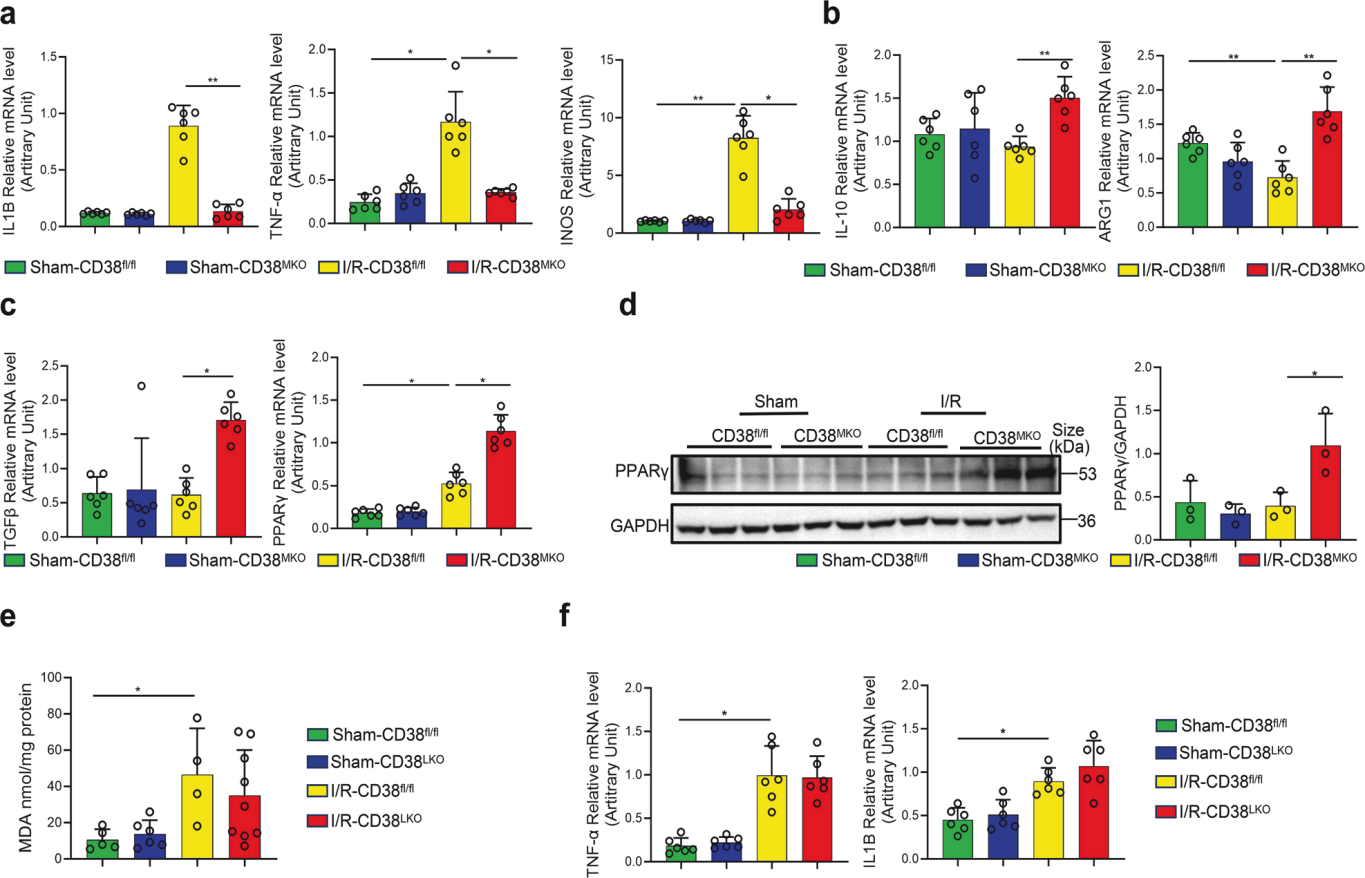
Myeloid CD38 deficiency reduced hepatic ischemia-reperfusion-triggered inflammation. **a**–**c** The mRNA expressions of IL1B, TNF-α, iNOS, IL-10, ARG1, TGF-β and PPARγ were analyzed by QPCR in CD38^MKO^ and CD38^fl/fl^ mice after HIRI. **d** The expressions and the quantitative analysis of PPARγ were detected by Western blot in liver tissues of CD38^MKO^ and CD38^fl/fl^ mice after HIRI. **e** MDA contents were measured in liver tissues of CD38^LKO^ and CD38^fl/fl^ mice after HIRI. **f** The mRNA expressions of TNF-α and IL1B were determined by QPCR in liver tissues of CD38^LKO^ and CD38^fl/fl^ mice after HIRI. Data are shown as means ± SEM, *p < 0.05, **p < 0.01 and ***p < 0.001, *n* = 3 ~ 9 per group

Oxidative stress, resulting in excessive reactive oxygen species, is a major contributor to HIRI.^25^ To elucidate mechanisms by which CD38 deficiency is protective against HIRI, we measured superoxide anion contents, malondialdehyde (MDA) contents that represent a product of lipid peroxidation, and oxidative stress-related gene expressions in the liver tissues of CD38-deficient mice. The results showed that CD38 deficiency significantly inhibited the production of superoxide anion (Fig. 2a) and MDA (Fig. 2b), promoted SOD2 expression, and declined NOX2 (Fig. 2d) expression in liver tissues of CD38^KO^ mice compared to CD38^fl/fl^ mice under HIRI conditions. In addition, ELISA assays showed that CD38 deficiency remarkably reduced the levels of serum TNF-α and IL-1β (Fig. 2c) in CD38^KO^ mice. The results from qRT-PCR also indicated that CD38 deficiency markedly reduced the expressions of TNF-α and IL1B, and enhanced the expressions of TGF-β, CD206 and PPARγ in liver tissues of CD38^KO^ mice (Fig. 2e). Furthermore, western blot analysis confirmed that IL-1β expression (Fig. 2f) was reduced, while PPARγ expression (Fig. 2g) was elevated in liver tissues of CD38^KO^ mice during HIRI. These results indicated that CD38 deficiency alleviated HIRI-induced oxidative stress and inflammation in liver tissues. Next, we further investigated the roles of macrophagic CD38 in HIRI-triggered hepatic inflammation. Our results revealed that the mRNA levels of pro-inflammatory markers IL1B, TNF-α, and iNOS (Fig. 3a) were greatly decreased, whereas the mRNA expressions of IL-10, ARG1, TGF-,β and PPARγ (Fig. 3b, c) were notably elevated in CD38^MKO^ mice compared to CD38^fl/fl^ mice following HIRI. Western blotting results also confirmed that the expression of PPARγ was markedly elevated in liver tissues of CD38^MKO^ mice after HIRI (Fig. 3d). In contrast, CD38 deletion in hepatocytes did not affect HIRI-induced increases of MDA formation (Fig. 3e) and the expressions of TNF-α and IL1B (Fig. 3f) in liver tissues of CD38^LKO^ mice. Taken together, these results indicated that CD38 depletion in innate immune cells, rather than hepatocytes, protected the liver against HIRI.

### Global and myeloid-specific deletion of CD38 reduces HIRI-induced pyroptosis in liver tissues

**Fig. 4:**
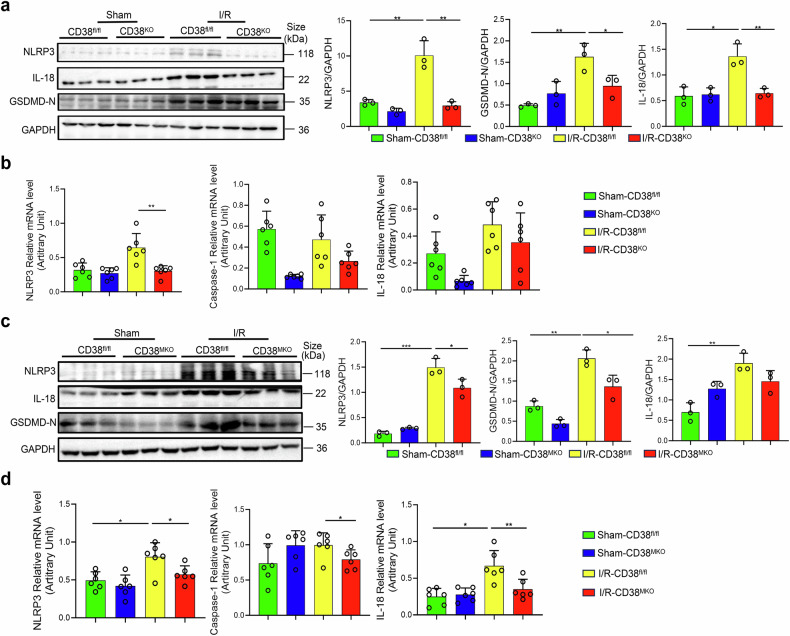
Global and myeloid deletion of CD38 ameliorated HIRI-induced pyroptosis in vivo. The protein expressions and the quantitative analysis of NLRP3, IL-18 and GSDMD-N and the mRNA expressions of NLRP3, Caspase-1 and IL-18 were examined by Western blot (**a**) and QPCR (**b**) in liver tissues of CD38^KO^ and CD38^fl/fl^ mice after HIRI, respectively. The protein expressions and the quantitative analysis of NLRP3, GSDMD-N and IL-18, and the mRNA expressions of NLRP3, Caspase-1 and IL-18 were determined by Western blot (**c**) and QPCR (**d**) in liver tissues from CD38^MKO^ and CD38^fl/fl^ mice after HIRI. Data are shown as means ± SEM, *p < 0.05, **p < 0.01and ***p < 0.001, *n* = 3–6 per group

Studies demonstrated that pyroptosis plays a critical role in HIRI.^22^ Our western blot analysis revealed that global CD38 deletion greatly suppressed HIRI-induced upregulation of NLRP3, IL-18, and cleaved GSDMD in liver tissues of CD38^KO^ mice compared to CD38^fl/fl^ mice (Fig. 4a). Additionally, qRT-PCR analysis showed that global deletion of CD38 remarkably inhibited HIRI-induced increases of the expressions of NLRP3, caspase 1 and IL-18 in liver tissues of CD38^KO^ mice (Fig. 4b). Interestingly, western blot results further revealed that myeloid-specific CD38 deletion markedly reduced the expressions of NLRP3, GSDMD-N, and IL-18 in liver tissues of CD38^MKO^ mice (Fig. 4c). Furthermore, our qPCR results also revealed that there was a great decline in the mRNA expressions of NLRP3, caspase-1, and IL-18 in CD38^MKO^ liver tissues in HIRI conditions (Fig. 4d). These results suggested that CD38 deficiency in innate immune cells was able to protect hepatocytes from HIRI via partially inhibiting pyroptosis.

### Myeloid CD38 deficiency protects hepatocytes from hypoxia/reoxygenation injury in vitro

**Fig. 5:**
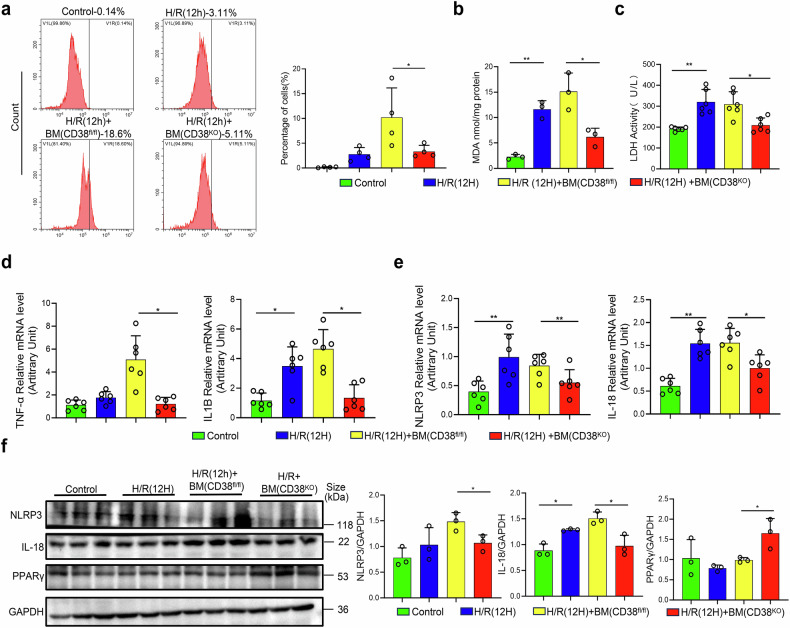
Myeloid CD38 deficiency alleviated hepatocytic hypoxia/reoxygenation injury in a co-culture condition in vitro. **a** Representative flow cytometry plots of PI staining, along with the quantitative analysis, for hepatocytes from CD38^fl/fl^ mice co-cultured with BMDMs from CD38^KO^ mice after H/R injury. MDA contents (**b**), LDH activities (**c**), the mRNA expressions of IL-1B (**d**), TNF-α (**d**), NLRP3 (**e**) and IL-18 (**e**), and the protein expressions and the quantitative analysis (**f**) of NLRP3, IL-18 and PPARγ were examined in primary hepatocytes from CD38^fl/fl^ mice co-cultured with BMDMs from CD38^KO^ mice after H/R injury. Data are shown as means ± SEM, *p < 0.05, **p < 0.01 and ***p < 0.001, *n* = 3–6 per group

To further investigate the protective effects of CD38 deletion in innate immune cells on HIRI, we established hypoxia/reoxygenation (H/R) model in vitro as previously described.^19^ BMDMs (Bone marrow-derived macrophages) from CD38^KO^ mice or CD38^fl/fl^ mice were co-cultured with primary hepatocytes from CD38^fl/fl^ mice under H/R conditions. FACS following PI staining indicated that H/R significantly reduced the viability of hepatocytes (Fig. 5a), which was further corroborated by microscopic observations (Supplementary Fig. 2a) and CCK-8 assay (Supplementary Fig. 2b). Meanwhile, H/R augmented MDA formation and LDH release (Fig. 5b, c). Conversely, CD38^KO^ mice-derived BMDMs efficiently abrogated the impacts of H/R on hepatocytes. Additionally, qRT-PCR analysis results revealed that H/R treatment markedly enhanced the expressions of IL1B and TNF-α, whereas BMDMs from CD38^KO^ mice reduced their expressions (Fig. 5d). Moreover, qRT-PCR analysis also showed that deletion of CD38 in BMDMs markedly inhibited H/R-induced increases of the expressions of genes involved in pyroptosis such as NLRP3 and IL-18 in hepatocytes (Fig. 5e). Furthermore, the western blot results confirmed that CD38 deficiency notably diminished the expressions of NLRP3 and IL-18, whereas it remarkably increased the expression of PPARγ, an important transcription factor in polarizing towards M2 type macrophages, in CD38^KO^ macrophage group treated with H/R (Fig. 5f). These results indicated that myeloid-specific deletion of CD38 had ability to protect hepatocytes from H/R injury in vitro via inhibiting pyroptosis and improving the polarization of M2 type macrophages. Furthermore, we conducted co-culture experiments by using bone marrow macrophages from CD38^LKO^ or CD38^MKO^ mice respectively with primary hepatocytes from CD38^fl/fl^ mice followed by H/R. Our results demonstrated that BMDMs from CD38^LKO^ mice exhibited a phenotype similar to that of BMDMs from CD38^fl/fl^ mice (Supplementary Fig. 2c, d) and the BMDMs from CD38^MKO^ mice displayed a phenotype resembling that of CD38^KO^ mice (Supplementary Fig. 2e). These results further explained that myeloid deletion of CD38 was able to protect hepatocytes from H/R injury in vitro via inhibiting pyroptosis and promoting the polarization of M2 type macrophages.

### Myeloid CD38 deficiency protects hepatocytes from ischemia-reperfusion injury through activating SIRT1-p53 pathway in macrophages

**Fig. 6:**
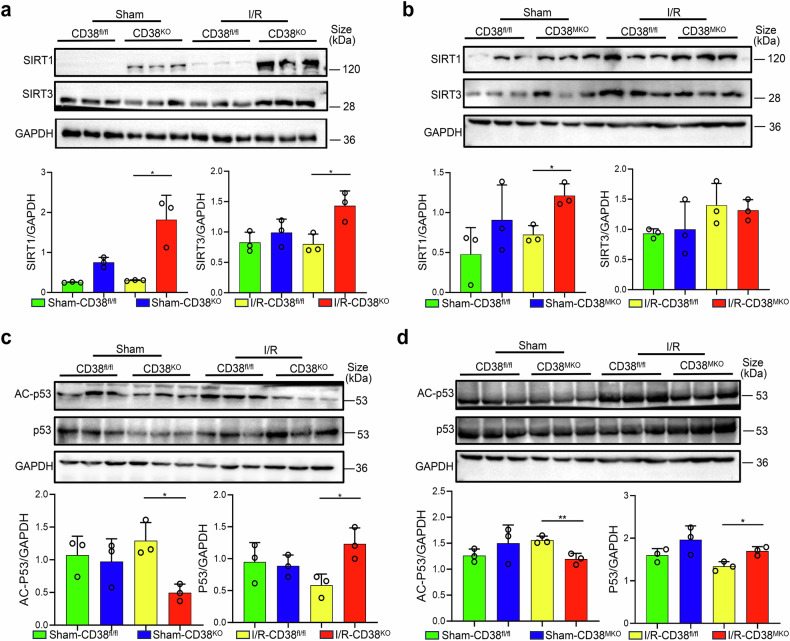
Global and myeloid deletion of CD38 upregulated SIRT1-p53 signaling pathway. The expressions and the quantitative analysis of SIRT1 (**a,** **b**), SIRT3 (**a,** **b**), p53 (**c,** **d**), and AC-p53 (**c,** **d**) were determined by Western blot in liver tissues from CD38^KO^, CD38^MKO^, and CD38^fl/fl^ mice after HIRI. Data are shown as means ± SEM, *p < 0.05, **p < 0.01 and ***p < 0.001, *n* = 3 per group

Sirtuin is a family of NAD^+^-dependent protein deacetylases that govern numerous target genes, such as p53 and PPARγ. It has been reported that hepatocyte-specific SIRT1 deficiency aggravated HIRI.^22^ Based on this, we examined the expressions of SIRT1 and SIRT3 in mouse liver tissues during HIRI. Western blot analysis revealed higher levels of both SIRT1 and SIRT3 proteins in CD38^KO^ liver tissues compared to CD38^fl/fl^ mice after HIRI (Fig. 6a). However, in CD38^MKO^ mice, only SIRT1 expression was increased, while SIRT3 remained unchanged (Fig. 6b). Moreover, we observed that p53 expression was greatly upregulated, while its acetylation level was notably reduced in liver tissues of CD38^KO^ (Fig. 6c) and CD38^MKO^ (Fig. 6d) mice compared with CD38^fl/fl^ mice after IR. Furthermore, CD38 deficiency markedly reduced the ratios of AC-P53/P53 in liver tissues in both CD38^KO^ and CD38^MKO^ mice compared with CD38^fl/fl^ mice (Supplementary Fig. 3a), and the concentrations of NAD^+^ were increased in liver tissues of CD38^KO^ mice, but not CD38^MKO^ mice compared with CD38^fl/fl^ mice (Supplementary Fig. 3b). In addition, qRT-PCR analysis indicated that the mRNA expressions of SIRT1 were higher in CD38^MKO^, but not CD38^LKO^ mice compared with CD38^fl/fl^ mice (Supplementary Fig. 3c), indicating that the protection of myeloid CD38 deficiency on HIRI might be related to activate SIRT1/p53 signaling pathway in macrophages, rather than directly to elevate the NAD^+^ concentration of hepatocytes.

### Myeloid CD38 deficiency promotes macrophage M2 polarization through activating NAD+/SIRT1 signaling pathways

**Fig. 7:**
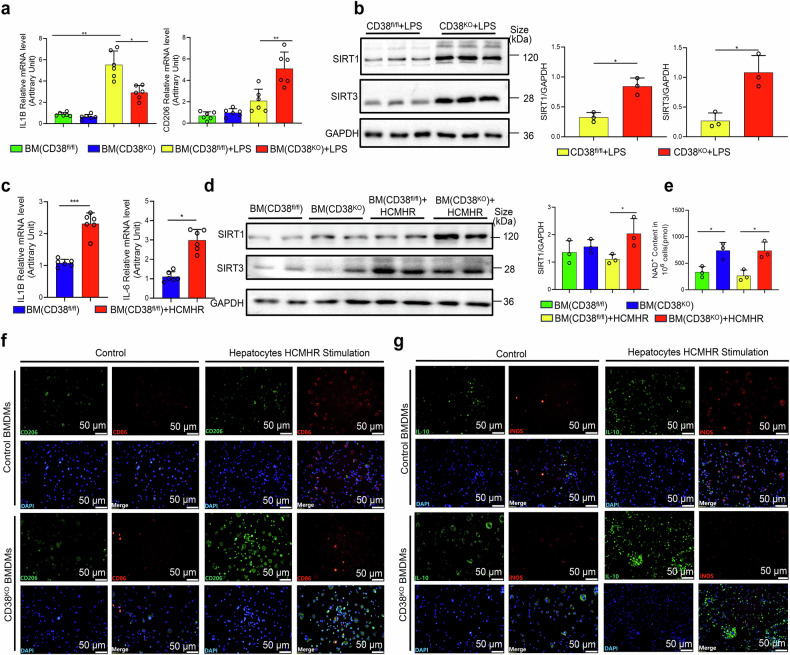
Myeloid CD38 deficiency promoted macrophage M2 polarization through activating NAD^**+**^/SIRT1 pathway in vitro. The mRNA expressions of IL1B (**a**), CD206 (**a**), and the protein expressions and the quantitative analysis of SIRT1 (**b**) and SIRT3 (**b**) were determined by QPCR and Western blot in BMDMs from CD38^fl/fl^ and CD38^KO^ mice after LPS stimulation, respectively. **c** The mRNA expressions of IL1B and IL-6 were determined by QPCR in BMDMs from CD38^fl/fl^ mice with the stimulation of hepatocytes-derived conditioned media after hypoxia/reoxygenation injury (HCMHR). The protein expressions and the quantitative analysis of SIRT1/SIRT3 (**d**), the NAD^+^ contents (**e**) and the infiltrations of the type 2 macrophages (CD206/Il-10, **f,** **g**), and type 1 macrophages (CD86/iNOS, **f,** **g**) were determined in BMDMs from CD38^KO^ and CD38^fl/fl^ mice with HCMHR stimulation. Data are shown as means ± SEM, *p < 0.05, **p < 0.01 and ***p < 0.001, *n* = 3–6 per group

To further investigate the role of CD38 in macrophage polarization, the expressions of the polarization-related genes were examined in LPS-stimulated BMDMs. Our qRT-PCR results showed that CD38 deficiency notably decreased IL1B expression and increased CD206 expression in CD38^KO^ BMDMs compared with CD38^fl/fl^ mice after stimulation with LPS (Fig. 7a). In addition, the expressions of SIRT1 and SIRT3 protein (Fig. 7b) and mRNA (Supplementary Fig. 3d and 3e) were significantly upregulated in BMDMs of CD38^KO^ mice with LPS stimulation. Furthermore, the mRNA expressions of IL1B and IL-6 were remarkably increased in BMDMs from CD38^fl/fl^ mice upon stimulation with hepatocytes-derived conditioned media after hypoxia/reoxygenation injury (HCMHR) (Fig. 7c). Moreover, SIRT1 expressions (Fig. 7d, Supplementary Fig. 3f) and NAD^+^ contents (Fig. 7e) were prominently augmented in BMDMs from CD38^KO^ mice compared to CD38^fl/fl^ mice after HCMHR stimulation although there was no obvious discrepancy in the expressions of SIRT3 (Supplementary Fig. 3g) in BMDMs of CD38^KO^ and CD38^fl/fl^ mice. Furthermore, our immunofluorescence analysis indicated that the activated macrophages were markedly increased in BMDMs of CD38^fl/fl^ mice after HCMHR stimulation (Fig. 7f, g). More importantly, in CD38^fl/fl^ mice, the activated macrophages were predominantly inflammatory M1 macrophages, characterized by CD86 or iNOS. In contrast, CD38^KO^ mice exhibited mainly anti-inflammatory M2 macrophages, denoted by CD206 or IL-10 (Fig. 7f, g). These results suggested that myeloid CD38 deficiency enhanced M2 macrophage polarization while inhibiting M1 macrophage polarization by activating the NAD^+^/SIRT1 pathway in vitro.

**Fig. 8:**
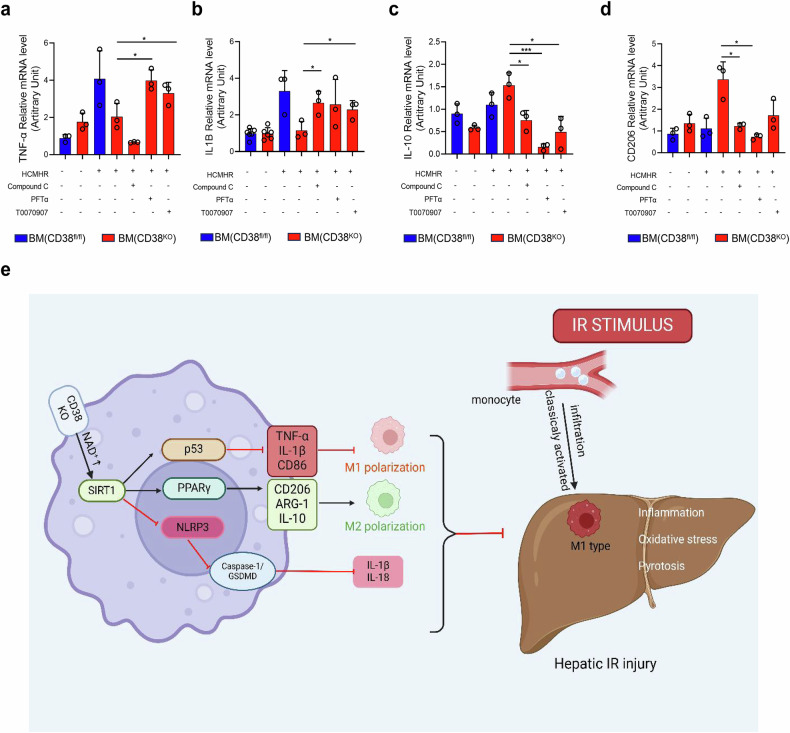
Myeloid CD38 deficiency facilitated macrophage M2-type polarization through activating SIRT1-p53 and SIRT1-PPARγ pathway in vitro*.* The mRNA expressions of TNF-α (**a**), IL1B (**b**), IL-10 (**c**) and CD206 (**d**) were determined by QPCR in BMDMs from CD38^KO^ and CD38^fl/fl^ mice with the stimulation of the hepatocytes-derived conditioned media after hypoxia/reoxygenation injury (HCMHR) in the pretreatment of Compound C (AMPK inhibitor), PFT-α (P53 inhibitor) and T0070907(PPARγ inhibitor), respectively. Data are shown as means ± SEM, *p < 0.05, **p < 0.01 and ***p < 0.001, *n* = 3 per group. **e** The mechanism of myeloid-specific deletion of CD38 (CD38^MKO^) protecting against hepatic ischemia/reperfusion injury (HIRI): CD38^MKO^ elevates the intracellular NAD^+^ levels in macrophages, and in turn, alleviates hepatic ischemia/reperfusion injury (HIRI)-induced inflammation and pyroptosis via activating SIRT1 signaling pathways in macrophages. On the one hand, SIRT1 promotes myeloid monocytes toward macrophage type 2 polarization through activating PPARγ signaling pathway in macrophages and inhibits monocytes toward macrophage type 1 polarization via activating p53 signaling, reducing HIRI-induced inflammation. On the other hand, SIRT1 also suppresses the release of pro-inflammatory factors such as IL1-β and IL18 through inactivating the canonical inflammasome-pyroptosis pathway of NLRP3-mediated caspase-1/GSDMD processing in macrophages. Image created with BioRender.com, with permission (agreement number: FG27ZL6X5O; citation to use: https://www.biorender.com/j12e628)

To delve deeper into the mechanism of myeloid CD38 deficiency in M2-type polarization of macrophages, we examined the gene expressions of SIRT1/p53 and SIRT1/PPARγ pathway utilizing specific inhibitors like Compound C (AMPK inhibitor), T0070907 (PPARγ inhibitor), and PFT-α (p53 inhibitor) in CD38^KO^ mice BMDMs after HCMHR stimulation. The outcomes demonstrated that all these inhibitors notably reversed HCMHR-induced downregulation of the levels of pro-inflammatory cytokines TNF-α (Fig. 8a) as well as IL1B (Fig. 8b), and upregulation of the levels of the anti-inflammatory markers IL-10 (Fig. 8c) along with CD206 (Fig. 8d) in BMDMs of CD38^KO^ mice. Our immunofluorescent staining results also revealed that these inhibitors led to a marked increase in CD86 and iNOS expression, and remarkably decreased the expressions of CD206 and IL-10 in BMDMs of CD38^KO^ mice with HCMHR stimulation (Supplementary Fig. 4a and 4b). These results indicated that myeloid CD38 deficiency facilitated macrophage M2-type polarization through activating CD38-SIRT1-PPARγ and CD38-SIRT1-p53 signaling pathways.

## Discussion

Hepatic ischemia-reperfusion injury (HIRI) presents a major complication in liver surgery and liver transplantation which stands as the main therapeutic choice in the end-stage liver disease. Nicotinamide adenine dinucleotide (NAD^+^) is a crucial co-enzyme that takes part in a wide range of biological processes and is regulated by a variety of transcription factors, as well as inflammatory mediators and cytokines.^26,27^ CD38 is the core enzyme to catalyze NAD^+^ degradation in mammalian cells. CD38 deficiency alleviated the senescence in cardiac cells, attenuated cardiac ischemia-reperfusion injury, improved oxidative stress induced by high-fat diet, and protected hepatocytes from nonalcoholic fatty liver disease.^17,18,28,29^ However, the impact of CD38 deficiency on HIRI has not been investigated yet. Previous studies have shown that HIRI-related macrophage activation and the subsequent release of inflammatory factors are the key factors contributing to graft dysfunction. Here, we reported that global or myeloid CD38 deficiency significantly alleviated HIRI through inhibiting oxidative stress, inflammation, and pyroptosis, suggesting that myeloid CD38 might participate in the process of HIRI. Currently, several approaches targeting macrophages have been reported.^30,31^ Our study demonstrated that CD38 silencing of myeloid macrophages exerted a remarkable protective effect against HIRI. Compared with directly targeting the other cells/tissues of the liver, altering the function of the circulating monocytes holds great promise for preventing and treating the HIRI. Therefore, our study indicates that myeloid CD38 might be a viable therapeutic target for preventing and treating HIRI in clinical scenarios.

One of the key causes of HIRI is oxidative stress. At the onset of IR, ROS bring about extensive harm to hepatocytes via lipid peroxidation, protein oxidation, mitochondrial impairment, and DNA damage. This is followed by monocyte and neutrophil invasion into the liver, leading to inflammation and ultimately hepatocyte apoptosis.^32^ In our study, we observed that global and myeloid CD38 deletion significantly reduced MDA formation in liver tissue or hepatocytes after HIRI in vivo or H/R injury in vitro, respectively. SOD2 is an antioxidant metalloenzyme for removing free radicals. We also found that myeloid and global CD38 deficiency upregulated the expression of SOD2, while significantly reducing the expression of NOX2, indicating that the protection of CD38 deficiency against HIRI was relevant to the inhibition of hepatic oxidative stress through macrophages.

Pyroptosis, a programmed cell death mode, is another important cause for HIRI. This process depends on inflammasomes to activate various caspases, leading to the cleavage and multimerization of gasdermin family proteins, including GSDMD, ultimately resulting in cell membrane perforation and death. It has been reported that myeloid-specific SIRT1 deficiency exacerbated pyroptosis via mediating the classical pyroptosis signaling pathway.^33^ In our study, the expressions of NLRP3, caspase-1, IL-18, and the cleaved GSDMD were markedly decreased in liver tissues of CD38^KO^ and CD38^MKO^ mice after HIRI. In vitro, our results also showed that myeloid-specific CD38 deficiency decreased the expressions of the genes involved in pyroptosis in hepatocytes under Hypoxia/Reoxygenation, indicating that CD38 deficiency reduced pyroptosis through inhibiting the NLRP3 signaling pathway-mediated increase of active form GSDMD-N expression.

Sirtuins, a family of NAD⁺-dependent deacetylases, have been reported to modulate circadian control and regulate various autoimmune diseases via epigenetic modification, chromatin remodeling, and other mechanisms.^34,35^ The increase of intracellular NAD^+^ levels caused by CD38 deletion directly affects the expression and activity of the sirtuin family of proteins. It has been reported that liver-specific SIRT1 deletion exacerbated HIRI through enhancing the XBP1/NLRP3 inflammatory pathway, whereas myeloid-specific SIRT1 deficiency increased pyroptosis.^22,33^ SIRT3 also played a role in alleviating HIRI by activating the downstream factors such as SOD2, CYP-D, and HIF1α to suppress ROS production.^36^ Recent studies showed that SIRT5 inhibited oxidative stress and inflammation by enhancing the expression of SOD1 and IDH2, thereby alleviating HIRI.^37^ In this study, we observed that SIRT1 and its target genes, PPARγ and p53, were notably upregulated in CD38^KO^ and CD38^MKO^ mice following HIRI. Our in vitro experiments also confirmed that CD38 deficiency alleviated HIRI through activating PPARγ and p53 signaling pathways. In addition, the effects of the macrophage-derived SIRT1 on chromatin remodeling and epigenetic modifications need to be further investigated in hepatocytes.

PPARγ is a key transcription factor for regulating M1/M2 type polarization in macrophages. PPARγ agonists suppress the secretion and expression of proinflammatory cytokines in human monocytes, inhibit M1 macrophage activation in vitro, and facilitate M2 macrophage differentiation.^38^ It has been reported that IL-4 and IL-13 regulate macrophage polarization towards M2 type through STAT6, which was regulated by the transcription factor PPARγ. In addition, activation of STAT6 elevated the expression and activity of PPARγ and then further enhanced macrophage M2 type polarization.^39^ p53, a tumor suppressor and transcription factor, also participated in macrophage polarization and pyroptosis. It was reported that myeloid-specific HO-1 deficiency reduced the expression and activity of SIRT1, resulting in diminished expression and acetylation of p53, exacerbating HIRI, whereas activation of SIRT1 reversed this process in mice.^21^ SIRT1 activation in macrophages also reduced target gene ASC expression by deacetylating p53, prevented activation and formation of NLRP3, and hence reduced pro-inflammatory cytokine secretion IL-1β and IL-18.^40^ In our study, we observed that the TNF-α and IL-1β expressions were significantly reduced, and the expressions of IL-10, CD206 and TGF-β were greatly enhanced in CD38^KO^ and CD38^MKO^ mice under HIRI condition. Additionally, we observed a great increase in total p53 and PPARγ expression, while the acetylation level of p53 was notably reduced. Moreover, the protective influence of CD38 deletion against H/R was reversed by using specific inhibitors to target p53, AMPK (the key signaling protein kinase in the PPARγ pathway), and PPARγ. These results indicated that CD38 deficiency activated PPARγ and p53 signaling pathways by upregulating SIRT1 to inhibit inflammatory cytokine secretion and promote macrophage M2 type polarization, thereby alleviating HIRI.

There are certain limitations to our research as well. First, although our data indicated that CD38 deletion in bone marrow-derived macrophages played a crucial protective role in hepatic IR, macrophages possess different activation phases due to plasticity and context-dependence and modification of tissue microenvironment.^15^ Macrophages may have different roles in various liver diseases or at different stages of the same disease, making clinical application complicated.^41^ The roles and mechanisms of macrophage polarization in liver diseases should be further investigated in the future. Second, it has been reported that although the bone marrow-derived macrophages were the dominating cell types of the infiltrating macrophage, Kupffer cells in the liver were significantly reduced during the HIRI. However, as the largest innate macrophage population in vivo, the role of Kupffer cells with CD38 deficiency in hepatic IR needs to be further investigated.^42,43^ Third, the process of HIRI is a dynamic process with different pathological changes at different times after ischemia and reperfusion. In our study, we focused solely on a systematic investigation of 1-hour ischemia followed by 12-hour reperfusion, and the other time points need to be further observed.

We previously observed that there was a protective effect of global CD38 deletion on cardiac ischemia-reperfusion and nonalcoholic fatty liver disease (NAFLD).^19^ It has been reported that CD38 deficiency upregulated IL-1β/TLR4/NLRP3/GSDMD signaling axis via the TLR1/ERK/NF-κB pathway, resulting in aggravated liver injury in the sepsis model of CD38^KO^ mice.^28,43^ In our study, we demonstrated that myeloid-specific CD38 deletion greatly ameliorated HIRI in mice, strengthening the view that functional regulation of CD38/NAD^+^/SIRT1 axis may improve aseptic inflammation and tissue damage.

In summary, our study demonstrated that CD38 deficiency, especially in myeloid cells, improved hepatic I/R or H/R injury both in vivo and in vitro by suppressing pyroptosis, oxidative stress and inflammation (Fig. 8e). The absence of myeloid CD38 not only enhanced M2 polarization of macrophages and inhibited M1 polarization, but also reduced NLRP3-mediated pyroptosis through activation of NAD^+^/SIRT1 pathway in macrophages, which in turn, led to the inhibition of hepatic oxidative stress, inflammation, and pyroptosis, eventually protecting against HIRI. Certainly, our study should provide an insight in elucidating the mechanism of HIRI, and CD38 of macrophages might be a potential therapeutic target for prevention and treatment of HIRI clinically.

## Materials and methods

### Study approval

All animal experiments described herein were reviewed and approved by the Nanchang University Ethics Committee (Approval Code: X20200630) and were conducted according to Jiangxi Province Laboratory Animal Care Guidelines for animal research. All mice were backcrossed into C57BL/6 background at least 6 generations before experiments.

### Animals

CD38^fl/fl^ mice were generated by Cyagen (SuZhou, China). Global CD38 knockout (CD38^KO^), myeloid-specific CD38 knockout (CD38^MKO^), and liver/hepatocyte-specific CD38 knockout (CD38^LKO^) mice were obtained by crossing CD38^fl/fl^ mice with EIIa-Cre mice (Cyagen, China), lysM (lysozyme M)-Cre mice and Alb (Albumin)-Cre mice, respectively. The mice used in the study were 6-8 weeks old, and the age-matched CD38^fl/fl^ mice served as controls.

### HIRI model in vivo

A HIRI mouse model was developed based on a previously established protocol.^44^ Briefly, a nontraumatic vascular clamp was applied to the portal vein branches to temporarily block blood flow of the left and middle liver lobes. After 1 hour of ischemia, the clamp was removed to restore blood circulation, followed by 12 hours of reperfusion. The mice were then sacrificed, and serum, tissues, and organs were collected for further analysis.

### H/R model in vitro

Cell H/R model was established with ref. ^19^. Briefly, after cell adhesion, the culture medium was swapped out for a low-serum medium with 1% FBS. After culturing for hours, the cells were moved to a three-gas incubator (0.5% O₂, 5% CO₂, 37°C) for 12 hours of hypoxia, and then moved to a normal incubator (5% CO_2_, 37 °C) for reoxygenation cultivation for 12 hours.

### Isolation and extraction of cells

Primary hepatocytes and bone marrow-derived macrophages (BMDMs) were harvested from male mice aged 6 to 8 weeks, following established protocols.^45,46^ In short, the mice were anesthetized and securely positioned on a sterilized surgical tray. The hepatic portal vein and inferior vena cava were then carefully exposed. A D-Hanks solution was introduced into the liver via the hepatic portal vein, ensuring the organ was fully saturated. Once the outflow fluid from the incised inferior vena cava transitioned from a reddish hue to a clear appearance, the perfusion process was halted. Then the perfusion solution was replaced with a solution containing type IV collagen to continuously perfuse the liver, and then stopped perfusion when the liver was soft and inelastic. The liver was removed and put into 4 °C pre-cooled DMEM medium. Then the liver envelope was torn with forceps and the liver tissue was bluntly separated. To wrap up the process, the hepatocyte mixture was passed through a 200-mesh cell strainer. Afterward, the suspension was spun in a centrifuge at 300 rpm for five minutes. Once the supernatant was carefully removed, the remaining cell pellet was mixed again using DMEM medium. Finally, the cells were carefully transferred into a well plate that had been pre-coated with porcine skin collagen. Once they adhered, the medium was refreshed.

BMDMs were isolated from femurs and tibias of mice of each group. The BMDMs were subjected to treatment with red blood cell lysis buffer composed of 155 mM NH₄Cl, 12 mM NaHCO₃, and 0.1 mM EDTA, following which they were suspended in 2 ml of unsupplemented DMEM. Next, BMDMs were transferred into DMEM containing 10% fetal calf serum and M-CSF and cultured for 7 days with fresh medium being added on the 4^th^ day and the 6^th^ day.

### Cell culture and treatment

A co-culture system was set up using 0.4 μm TRASWELL chamber. Hepatocytes were positioned within the lower compartment, while bone marrow-derived macrophages (BMDMs) were placed in the upper one. The cell culture medium employed was DMEM (Dulbecco’s Modified Eagle’s Medium) sourced from Gibco (Grand Island, NY, USA), which was fortified with 10% fetal bovine serum (FBS) and 1% penicillin/streptomycin, both also obtained from Gibco. These cells were incubated at 37°C in a humidified environment with 5% CO₂. Prior to initiating the hypoxia/reoxygenation culture, the medium was switched to a low-serum formulation containing 1% FBS. After 4 hours, the cells were placed in a pre-set three gas incubator (0.5% O_2_, 5% CO_2_, 37 °C) for hypoxia culture for 12 hours, and then transferred to a common incubator (5% CO_2_, 37 °C) for reoxygenation culture for 12 hours. The inhibitors including Compound C (AMPK inhibitor, BML-275, MedChemExpress), PFTα (P53 inhibitor, HY-15484, MedChemExpress), T0070907 (PPARγ inhibitor, HY-13202, MedChemExpress) were added before conducting hypoxia/reoxygenation. The supernatant and cells were harvested for subsequent analysis.

### Measurements of multiple indices

MDA content and SOD activity were measured using corresponding assay kits (TBA method, Nanjing Jiancheng Bioengineering Institute, Nanjing, China), respectively. Briefly, cells or tissues were collected, homogenized, or broken by sonication. According to the instructions, the absorbances were measured at 532 (MDA) and 450 (SOD) nm wavelength using a microplate reader, respectively. The outcomes were adjusted for total protein levels, with every trial conducted at least three times to ensure reliability. For mouse blood samples, both whole blood and serum were collected, and the measurements of the complete blood count and analysis of liver function (AST, ALT) were conducted by Wuhan Servicebio Technology Co., Ltd.

### Haematoxylin and eosin (H&E) and DHE staining

Liver tissue was collected and preserved by fixation in 4% paraformaldehyde (PFA) for a full 24 hours. The tissue blocks were sectioned into 5 μm slices and treated with H&E or DHE following standard protocols.

### Flow cytometric analysis for propidium iodide (PI) staining

Following the established protocol, the cells were plated and incubated under standard conditions. Following the initial treatment, the cells were thoroughly washed and then suspended in phosphate-buffered saline (PBS). To facilitate staining, a 5 μL volume of propidium iodide (PI) solution was added to the cell mixture. Subsequently, the prepared samples were analyzed using the CytoFLEXLX system by Beckman Coulter for detailed flow cytometry.

### Immunofluorescence (IF) staining

The preparation of animal tissue sections was carried out in the same manner as previously described. A suspension of cells (4 × 10⁵ cells/mL) was plated onto coverslips and then treated with 4% paraformaldehyde (PFA) for 20 minutes at ambient temperature. After an extensive wash with PBS, the cells underwent permeabilization using 0.25% Triton X-100 for an hour, followed by a two-hour blocking step with 10% goat serum. The samples were then subjected to overnight immunostaining at 4 °C with monoclonal antibodies targeting IL-1β (GB12115, Servicebio), F4/80 (GB113373, Servicebio), ASGR1 (EPR22642, Abcam), CD86 (NBP2-25208, Novus Biologicals), CD206 (18704-1-AP, Proteintech), iNOS (PTM-6217, Jingjie), and IL-10 (PTM-5750, Jingjie). Following incubation with Alexa Fluor-tagged secondary antibodies (1:200 dilution) for one hour, the final images were obtained using fluorescence microscopy.

### Western blotting

Liver samples were homogenized in RIPA buffer containing 1 mM PMSF, and their protein concentrations were determined using a BCA assay kit. The lysates were then separated by SDS-PAGE and transferred onto PVDF membranes. To prevent nonspecific interactions, the membranes were blocked with 5% nonfat milk for one hour before being incubated with the appropriate antibodies overnight at 4 °C. CD38 (R&D), GAPDH (KangChen), SOD2 (CST), NOX2 (Abcam), IL-1β (CST), PPARγ (Santa Cruz Biotechnology), NLRP3 (CST), IL-18 (Abmart), GSDMD-N (Abmart), SIRT3 (Sigma-Aldrich), SIRT1 (Sigma-Aldrich), p53 (Abcam), Acetyl-p53 (Abcam) antibodies were diluted 1:400/1000, respectively. The membrane was then treated with an HRP-linked secondary antibody, diluted to a ratio of 1:5000, and left to incubate for one hour at room temperature. Detection was subsequently carried out using an ECL system, manufactured by Fdbio Science in Hangzhou, China.

### Quantitative real time PCR analysis (qRT-PCR)

RNA was isolated from both tissue samples and cell cultures using Trizol reagent (Invitrogen, Carlsbad, CA, USA). To quantify the RNA, a Nano 2000 spectrophotometer (Thermo Fisher) was employed. Following this, the RNA was reverse transcribed into cDNA using the Takara High Capacity cDNA Synthesis Kit (TaKaRa, Dalian, China), adhering strictly to the protocol provided by the manufacturer. The synthesized cDNA was then stored at −80°C for future use. Quantitative PCR was carried out on an ABI-ViiA7 PCR system. To assess mRNA expression levels, the ∆Ct method was applied, with GAPDH serving as the housekeeping gene for normalization. The specific primers utilized in the real-time PCR process are detailed as follows: GAPDH (F-AGCCAAAAGGGTCATCATCT; R-GGGGCCATCCACAGTCTTCT), CD38 (F-CTGCCAGGATAACTACCGACCT; R-CTTTCCCGACAGTGTTGCTTCT); IL1B (F-TGTAATGAAAGACGGCACACC; R-TCTTCTTTGGGTATTGCTTGG); IL-6 (F-GGAAATCGTGGAAATGAG; R-GCTTAGGCATAACGCACT); TNF-α (F-GTGGAACTGGCAGAAGAGGCA; R-AGAGGGAGGCCATTTGGGAAC); TGF-β (F-TACCTGAACCCGTGTTGCTCTC; R-GTTGCTGAGGTATCGCCAGGAA); CD206 (F-TAGCACTGGGTTGCATTGGT; R-TGCAGGGTTGACATGAGACC); PPARγ (F-TGCCAGTTTCGATCCGTAGA; R-ATGAATCCTTGGCCCTCTGA);iNOS (F-CTGCAGCACTTGGATCAGGAACCTG; R- GGAGTAGCCTGTGTGCACCTGGAA); IL-10(F-GCTCTTACTGACTGGCATGAG; R-CGCAGCTCTAGGAGCATGTG); NLRP3 (F-AACAGCCACCTCACTTCCAG; R-CCAACCACAATCTCCGAATG); Caspase-1 (F-GCACAAGACCTCTGACAGCA; R-TTGGGCAGTTCTTGGTATTC); IL-18 (F-GAAAATTTCAACTCTCTCCTGTG; R-CCTTCGTATGATGAAGATTCAAA).

### Statistics

The results are presented as mean values ± standard error of the mean (SEM). For comparing two groups, the Student’s t-test was employed, whereas one-way ANOVA was utilized for analyses involving multiple groups. Statistical significance was indicated with asterisks as follows: *p < 0.05, **p < 0.01, and ***p < 0.001.

## Supplementary information

Supplementary data

## Data Availability

The complete dataset from this study is accessible within the manuscript and its supplementary materials. Additionally, interested parties may request further information by contacting the corresponding author, provided the request is reasonable and justified.

## References

[1] Xiao, J. et al. Global liver disease burdens and research trends: Analysis from a Chinese perspective. *J. Hepatol.* **71**, 212–221 (2019). 30871980 10.1016/j.jhep.2019.03.004

[2] Kadono, K. et al. SIRT1 regulates hepatocyte programmed cell death via GSDME - IL18 axis in human and mouse liver transplantation. *Cell Death Dis.* **14**, 762 (2023). 37996424 10.1038/s41419-023-06221-0 PMC10667508

[3] Hirao, H., Nakamura, K. & Kupiec-Weglinski, J. W. Liver ischaemia-reperfusion injury: a new understanding of the role of innate immunity. *Nat. Rev. Gastroenterol. Hepatol.* **19**, 239–256 (2022). 34837066 10.1038/s41575-021-00549-8

[4] Ocuin, L. M. et al. Nilotinib protects the murine liver from ischemia/reperfusion injury. *J. Hepatol.* **57**, 766–773 (2012). 22641092 10.1016/j.jhep.2012.05.012 PMC3437237

[5] Li, F. et al. The protective effect of PNU-282987, a selective α7 nicotinic acetylcholine receptor agonist, on the hepatic ischemia-reperfusion injury is associated with the inhibition of high-mobility group box 1 protein expression and nuclear factor κB activation in mice. *Shock* **39**, 197–203 (2013). 23324890 10.1097/SHK.0b013e31827aa1f6

[6] Duffy, J. P. et al. Long-term patient outcome and quality of life after liver transplantation: analysis of 20-year survivors. *Ann. Surg.* **252**, 652–661 (2010). 20881772 10.1097/SLA.0b013e3181f5f23a

[7] Zhang, S. et al. Endothelial YAP/TEAD1-CXCL17 signaling recruits myeloid-derived suppressor cells against liver ischemia-reperfusion injury. *Hepatology* **81**, 888–902 (2025). 38407233 10.1097/HEP.0000000000000773 PMC11825485

[8] Miao, L. et al. Extracellular vesicles containing GAS6 protect the liver from ischemia-reperfusion injury by enhancing macrophage efferocytosis via MerTK-ERK-COX2 signaling. *Cell Death Discov.* **10**, 401 (2024). 39256347 10.1038/s41420-024-02169-y PMC11387478

[9] Deng, R. M. & Zhou, J. Targeting NF-κB in Hepatic Ischemia-Reperfusion Alleviation: from Signaling Networks to Therapeutic Targeting. *Mol. Neurobiol.* **61**, 3409–3426 (2024). 37991700 10.1007/s12035-023-03787-w

[10] Franz, S., Ertel, A., Engel, K. M., Simon, J. C. & Saalbach, A. Overexpression of S100A9 in obesity impairs macrophage differentiation via TLR4-NFkB-signaling worsening inflammation and wound healing. *Theranostics* **12**, 1659–1682 (2022). 35198063 10.7150/thno.67174 PMC8825590

[11] Takeuchi, D. et al. Interleukin 18 causes hepatic ischemia/reperfusion injury by suppressing anti-inflammatory cytokine expression in mice. *Hepatology* **39**, 699–710 (2004). 14999688 10.1002/hep.20117

[12] Ye, L. et al. Effect of hepatic macrophage polarization and apoptosis on liver ischemia and reperfusion injury during liver transplantation. *Front. Immunol.* **11**, 1193 (2020). 32676077 10.3389/fimmu.2020.01193 PMC7333353

[13] Zhang, M. et al. Myeloid HO-1 modulates macrophage polarization and protects against ischemia-reperfusion injury. *JCI insight* **3**, 120596 (2018). 30282830 10.1172/jci.insight.120596 PMC6237471

[14] Li, S. L. et al. Liraglutide Attenuates Hepatic Ischemia-Reperfusion Injury by Modulating Macrophage Polarization. *Front Immunol.* **13**, 869050 (2022). 35450076 10.3389/fimmu.2022.869050 PMC9016191

[15] Shapouri-Moghaddam, A. et al. Macrophage plasticity, polarization, and function in health and disease. *J. Cell Physiol.* **233**, 6425–6440 (2018). 29319160 10.1002/jcp.26429 PMC13482560

[16] Piedra-Quintero, Z. L., Wilson, Z., Nava, P. & Guerau-de-Arellano, M. CD38: An immunomodulatory molecule in inflammation and autoimmunity. *Front Immunol.* **11**, 597959 (2020). 33329591 10.3389/fimmu.2020.597959 PMC7734206

[17] Guan, X. H. et al. CD38 deficiency protects the heart from ischemia/reperfusion injury through activating SIRT1/FOXOs-mediated antioxidative stress pathway. *Oxid. Med Cell Longev.* **2016**, 7410257 (2016). 27547294 10.1155/2016/7410257 PMC4983367

[18] Wang, L. F. et al. CD38 deficiency protects heart from high fat diet-induced oxidative stress via activating Sirt3/FOXO3 pathway. *Cell Physiol. Biochem* **48**, 2350–2363 (2018). 30114710 10.1159/000492651

[19] Xie, L. et al. CD38 deficiency protects mice from high fat diet-induced nonalcoholic fatty liver disease through activating NAD(+)/Sirtuins signaling pathways-mediated inhibition of lipid accumulation and oxidative stress in hepatocytes. *Int J. Biol. Sci.* **17**, 4305–4315 (2021). 34803499 10.7150/ijbs.65588 PMC8579443

[20] Jablonski, K. A. et al. Novel markers to delineate murine M1 and M2 macrophages. *PLoS One* **10**, e0145342 (2015). 26699615 10.1371/journal.pone.0145342 PMC4689374

[21] Xu, B. et al. Metabolic rewiring of Kynurenine pathway during hepatic ischemia-reperfusion injury exacerbates liver damage by impairing NAD homeostasis. *Adv. Sci. (Weinh.)* **9**, e2204697 (2022). 36310151 10.1002/advs.202204697 PMC9762284

[22] Li, F. et al. SIRT1 alleviates hepatic ischemia-reperfusion injury via the miR-182-mediated XBP1/NLRP3 pathway. *Mol. Ther. Nucleic Acids* **23**, 1066–1077 (2021). 33664991 10.1016/j.omtn.2020.11.015 PMC7887305

[23] Nakamura, K. et al. Sirtuin 1 attenuates inflammation and hepatocellular damage in liver transplant ischemia/Reperfusion: From mouse to human. *Liver Transpl.* **23**, 1282–1293 (2017). 28719070 10.1002/lt.24821 PMC5705033

[24] He, J. et al. Myeloid deletion of Cdc42 protects liver from hepatic ischemia-reperfusion injury via inhibiting macrophage-mediated inflammation in mice. *Cell Mol. Gastroenterol. Hepatol.* **17**, 965–981 (2024). 38342302 10.1016/j.jcmgh.2024.01.023 PMC11047801

[25] Ji, H. et al. Neuropeptide PACAP in mouse liver ischemia and reperfusion injury: immunomodulation by the cAMP-PKA pathway. *Hepatology* **57**, 1225–1237 (2013). 22532103 10.1002/hep.25802 PMC3479352

[26] Malavasi, F. et al. Evolution and function of the ADP ribosyl cyclase/CD38 gene family in physiology and pathology. *Physiol. Rev.* **88**, 841–886 (2008). 18626062 10.1152/physrev.00035.2007

[27] Chini, E. N., Chini, C. C. S., Espindola Netto, J. M., de Oliveira, G. C. & van Schooten, W. The pharmacology of CD38/NADase: An emerging target in cancer and diseases of aging. *Trends Pharm. Sci.* **39**, 424–436 (2018). 29482842 10.1016/j.tips.2018.02.001 PMC5885288

[28] Du, Y. et al. CD38 deficiency up-regulated IL-1β and MCP-1 through TLR4/ERK/NF-κB pathway in sepsis pulmonary injury. *Microbes Infect.* **23**, 104845 (2021). 34098107 10.1016/j.micinf.2021.104845

[29] Wang, L. F. et al. CD38 deficiency alleviates D-Galactose-induced myocardial cell senescence through NAD(+)/Sirt1 signaling pathway. *Front Physiol.* **10**, 1125 (2019). 31551807 10.3389/fphys.2019.01125 PMC6735286

[30] Chen, Y. et al. Macrophage-specific in vivo RNA editing promotes phagocytosis and antitumor immunity in mice. *Sci. Transl. Med.* **17**, eadl5800 (2025). 39813319 10.1126/scitranslmed.adl5800

[31] Uehara, K. et al. Targeted delivery to macrophages and dendritic cells by chemically modified mannose ligand-conjugated siRNA. *Nucleic Acids Res.* **50**, 4840–4859 (2022). 35524566 10.1093/nar/gkac308 PMC9122583

[32] Bardallo, R. G. et al. Nrf2 and oxidative stress in liver ischemia/reperfusion injury. *FEBS J.* **289**, 5463–5479 (2022). 34967991 10.1111/febs.16336

[33] Kadono, K. et al. Myeloid Ikaros-SIRT1 signaling axis regulates hepatic inflammation and pyroptosis in ischemia-stressed mouse and human liver. *J. Hepatol.* **76**, 896–909 (2022). 34871625 10.1016/j.jhep.2021.11.026 PMC9704689

[34] Nakahata, Y. et al. The NAD+-dependent deacetylase SIRT1 modulates CLOCK-mediated chromatin remodeling and circadian control. *Cell* **134**, 329–340 (2008). 18662547 10.1016/j.cell.2008.07.002 PMC3526943

[35] Tao, Z., Jin, Z., Wu, J., Cai, G. & Yu, X. Sirtuin family in autoimmune diseases. *Front. Immunol.* **14**, 1186231 (2023). 37483618 10.3389/fimmu.2023.1186231 PMC10357840

[36] Katwal, G. et al. SIRT3 a Major Player in Attenuation of Hepatic Ischemia-Reperfusion Injury by Reducing ROS via Its Downstream Mediators: SOD2, CYP-D, and HIF-1α. *Oxid. Med. Cell Longev.* **2018**, 2976957 (2018). 30538800 10.1155/2018/2976957 PMC6258096

[37] Zheng, D., Qiwen, Z., He, D., He, Y. & Yang, J. SIRT5 alleviates hepatic ischemia and reperfusion injury by diminishing oxidative stress and inflammation via elevating SOD1 and IDH2 expression. *Exp. Cell Res.* **419**, 113319 (2022). 35995176 10.1016/j.yexcr.2022.113319

[38] Daniel, B. et al. The nuclear receptor PPARγ controls progressive macrophage polarization as a ligand-insensitive epigenomic ratchet of transcriptional memory. *Immunity* **49**, 615–626.e616 (2018). 30332629 10.1016/j.immuni.2018.09.005 PMC6197058

[39] Linares, I. et al. PPAR-gamma activation is associated with reduced liver ischemia-reperfusion injury and altered tissue-resident macrophages polarization in a mouse model. *PLoS One* **13**, e0195212 (2018). 29617419 10.1371/journal.pone.0195212 PMC5884549

[40] Huang, Y. et al. Inflammasome activation and pyroptosis via a lipid-regulated SIRT1-p53-ASC axis in macrophages from male mice and humans. *Endocrinology* **163** (2022). 10.1210/endocr/bqac014 PMC8896164 35136993

[41] Krenkel, O. & Tacke, F. Liver macrophages in tissue homeostasis and disease. *Nat. Rev. Immunol.* **17**, 306–321 (2017). 28317925 10.1038/nri.2017.11

[42] Guillot, A. & Tacke, F. Liver macrophages: old dogmas and new insights. *Hepatol. Commun.* **3**, 730–743 (2019). 31168508 10.1002/hep4.1356 PMC6545867

[43] Wang, C. et al. Macrophage polarization and its role in liver disease. *Front Immunol.* **12**, 803037 (2021). 34970275 10.3389/fimmu.2021.803037 PMC8712501

[44] Zhai, Y. et al. Cutting edge: TLR4 activation mediates liver ischemia/reperfusion inflammatory response via IFN regulatory factor 3-dependent MyD88-independent pathway. *J. Immunol.* **173**, 7115–7119 (2004). 15585830 10.4049/jimmunol.173.12.7115

[45] Kamo, N., Ke, B., Busuttil, R. W. & Kupiec-Weglinski, J. W. PTEN-mediated Akt/β-catenin/Foxo1 signaling regulates innate immune responses in mouse liver ischemia/reperfusion injury. *Hepatology* **57**, 289–298 (2013). 22807038 10.1002/hep.25958 PMC3524373

[46] Czaya, B. et al. Induction of an inflammatory response in primary hepatocyte cultures from mice. *J. Vis. Exp.* **121**, 55319 (2017). 10.3791/55319 PMC5408960 28362385

