# Supplementary material for: Myeloid but not hepatocytic CD38 is a key driver for hepatic ischemia/reperfusion injury
Source: Signal Transduct Target Ther. 2025 May 9;10:150. doi: 10.1038/s41392-025-02233-8 (PMC12062225; doi:10.1038/s41392-025-02233-8)

Supplementary Materials for

**Myeloid but not hepatocytic CD38 is a key driver for hepatic ischemia/reperfusion injury**

Qi-Hang Zhao^1,2^, Ya-Ting Zhang^1,3^, Ke Wen^1^, Qi Ding^1^, Zi-Ying Chen^1^, Dilinuer Tula^1^, Jia-Hui Li^1^, Juan Zhou^4^, Yun-Fei Xiao^1^, Xiao-Hui Guan^1^, Ke-Yu Deng^1,2,3*^, Ling-Fang Wang^1*^, Hong-Bo Xin^1,2,3*^

**This PDF file includes:**

Figures. S1 to S5 and the original films of immunoblots



 Supplementary Fig.1 Validation of CD38 deletion and its effects on HIRI.

(**a**) The mRNA expressions of CD38 were examined by QPCR analysis in liver tissues of global CD38 knockout (CD38^KO^) mice compared with CD38^fl/fl^ mice after I/R injury. (**b**-**c**) The expressions of CD38 protein were determined by Western blot for evaluating the efficiency of CD38 deletion in primary hepatocytes (**b**) and bone marrow-derived macrophages (**c**) from CD38^fl/fl^ and hepatocyte-specific CD38 knockout (CD38^LKO^) mice or myeloid-specific CD38 knockout (CD38^MKO^) mice. (**d**) The activities of serum ALT and AST were examined in CD38^fl/fl^ and CD38^LKO^ mice subjected to hepatic ischemia/reperfusion injury. (**e**) The activities of serum LDH were examined in CD38^fl/fl^ and CD38^KO^ mice subjected to hepatic ischemia/reperfusion injury. (**f**) Representative H&E staining images and the quantitative analysis of liver ischemic necrosis were taken from CD38^LKO^ mice subjected to liver I/R injury, respectively. Data are shown as means ± SEM, *p<0.05, **p<0.01 and ***p<0.001, n = 3~9 per group





Supplementary Fig.2 Supplementary results of the H/R model co-culture System.

(**a**-**b**) Representative bright-field microscopy images (**a**) and the results of the CCK-8 assay (**b**) for hepatocytes from CD38^fl/fl^ mice co-cultured with BMDMs from CD38^KO^ or CD38^fl/fl^ mice after hypoxia/reoxygenation (H/R) injury. (**c**) LDH activities were examined in primary hepatocytes from CD38^flox/flox^ mice co-cultured with BMDMs from CD38^LKO^ or CD38^fl/fl^ mice after hypoxia/reoxygenation (H/R) injury. The mRNA expressions of TNF-α, IL-1β were examined in primary hepatocytes from CD38^fl/fl^ mice co-cultured with BMDMs from CD38^LKO^ (**d**) or CD38^MKO^ (**e**) mice after hypoxia/reoxygenation (H/R) injury. Data are shown as means ± SEM, *p<0.05, **p<0.01 and ***p<0.001, n = 6 per group.





Supplementary Fig.3 CD38 deficiency promotes the activation of the SIRT1 - p53 axis.

(**a**) The ratios of p53 and AC-p53 expressions were determined by Western blot in liver tissues from CD38^KO^, CD38^MKO^ mice compared with CD38^fl/fl^ mice after I/R injury. (**b**) The NAD^+^ contents were measured in liver tissues from CD38^KO^_,_ CD38^MKO^ and CD38^fl/fl^ mice after I/R injury. (**c**) The mRNA expressions of SIRT1 were analyzed by QPCR in CD38^MKO^ or CD38^LKO^ and CD38^fl/fl^ mice after HIRI. (**d**, **e**) The mRNA expressions of SIRT1 and SIRT3 were determined by QPCR in BMDMs from CD38^fl/fl^ and CD38^KO^ mice after LPS stimulation. (**f**, **g**) The mRNA expressions of SIRT1 and SIRT3 were determined by QPCR in BMDMs from CD38^fl/fl^ and CD38^KO^ mice with stimulation of the primary hepatocytes-derived conditioned media after hypoxia/reoxygenation (HCMHR). Data are shown as means ± SEM, *p<0.05, **p<0.01 and ***p<0.001, n = 3~9 per group.





Supplementary Fig.4 Inhibiting the SIRT1-p53 or SIRT1-PPARγ pathways can reduce the protective effects conferred by myeloid CD38 deficiency.

(**a**, **b**) The expressions of the type 2 macrophages (CD206/IL-10) and the type 1 macrophages (CD86/iNOS) were determined by immunofluorescence staining in BMDMs from CD38^KO^ mice and CD38^fl/fl^ mice with HCMHR stimulation in the pretreatment of Compound C, PFT-α and T0070907, respectively.


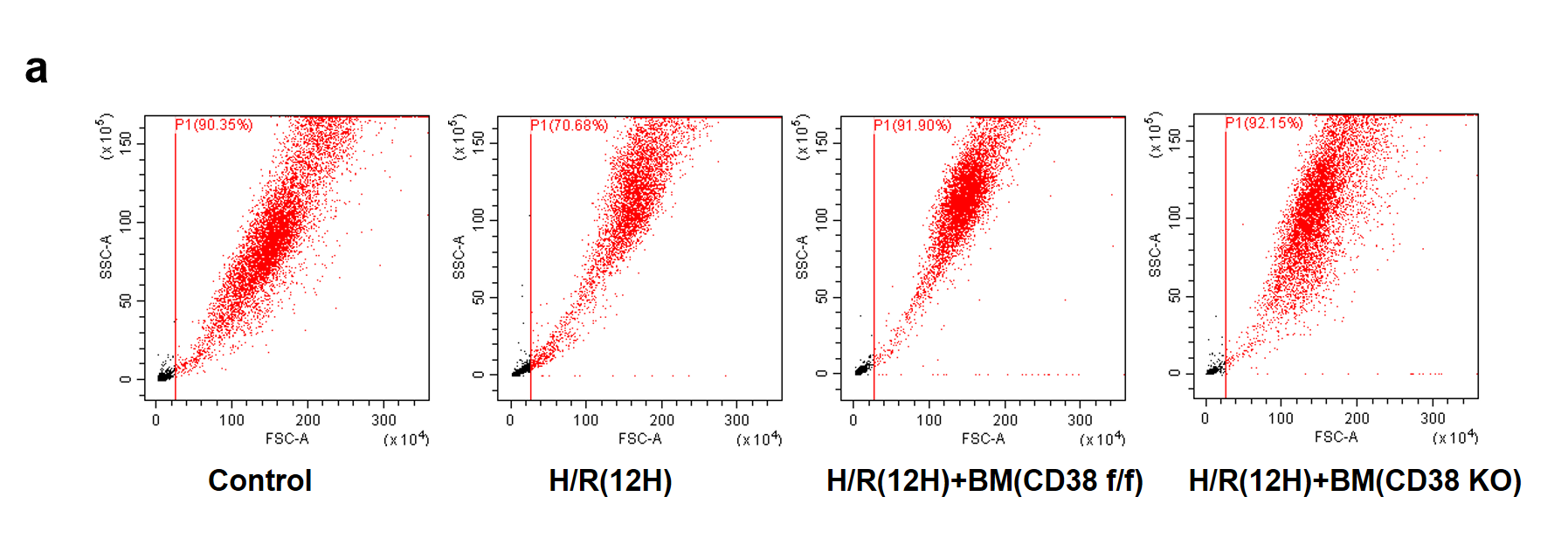


Supplementary Fig.5

(**a**) The gating strategies for **Fig 5a**.

**Original films of immunoblots**


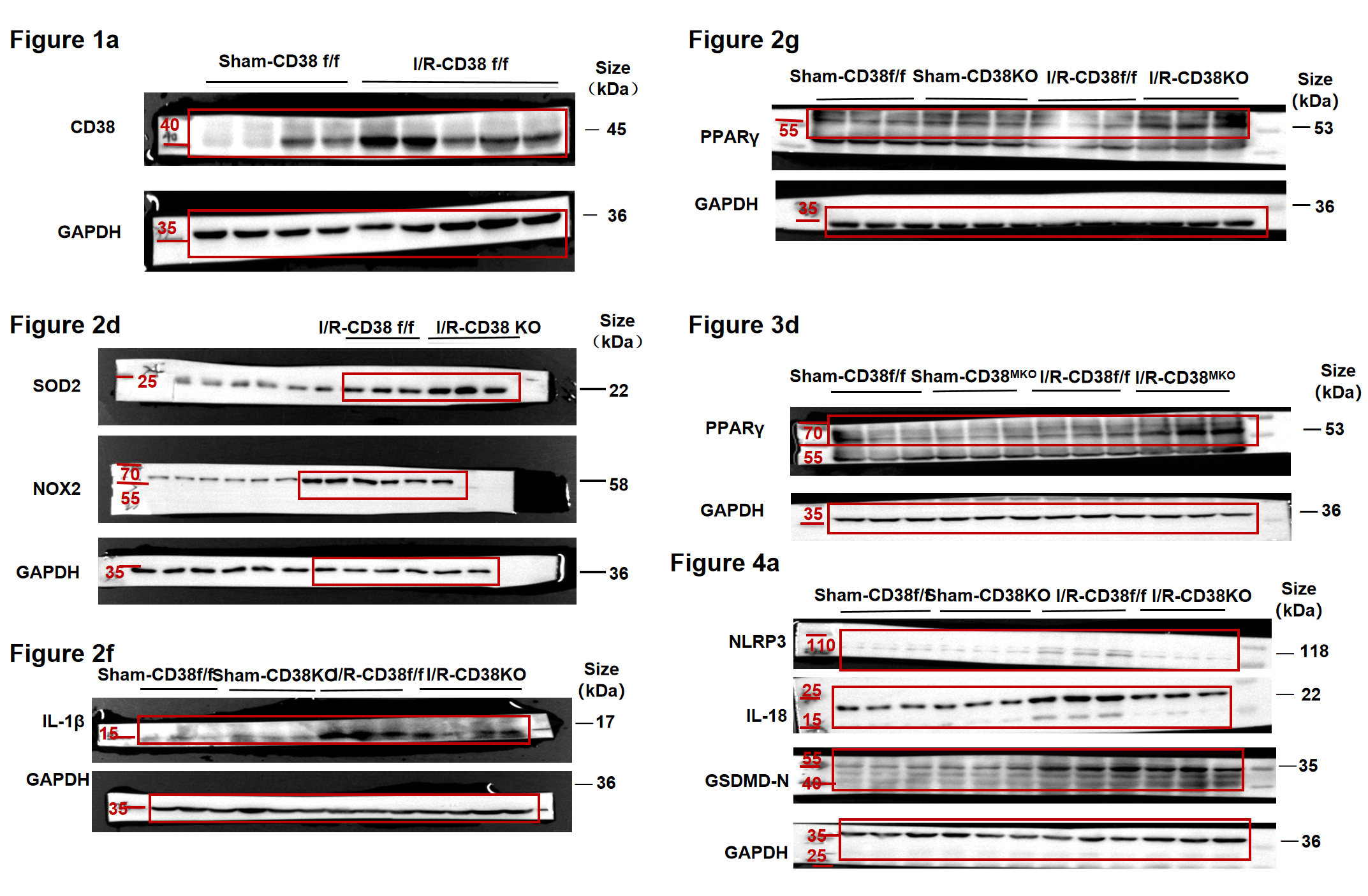


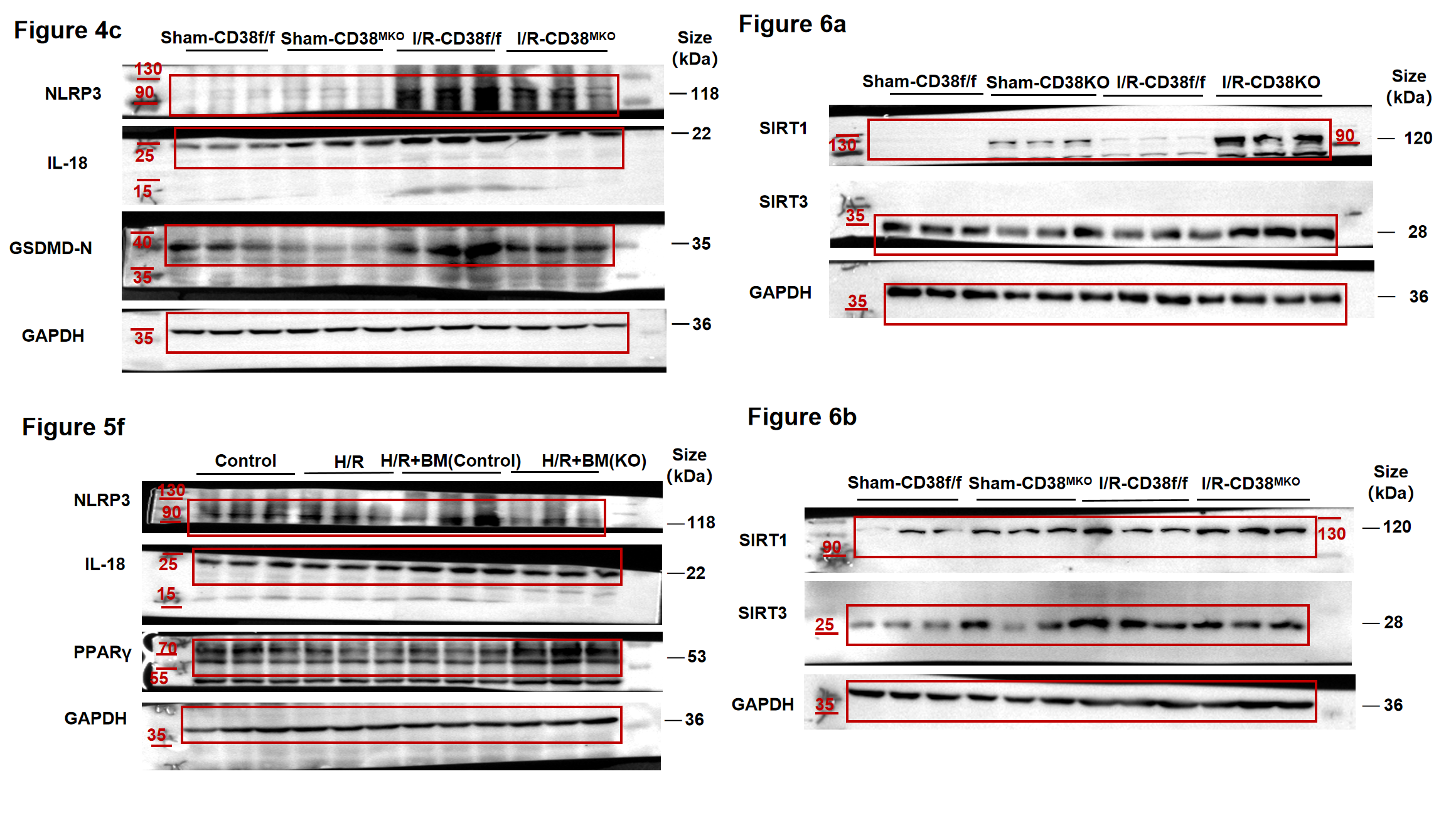


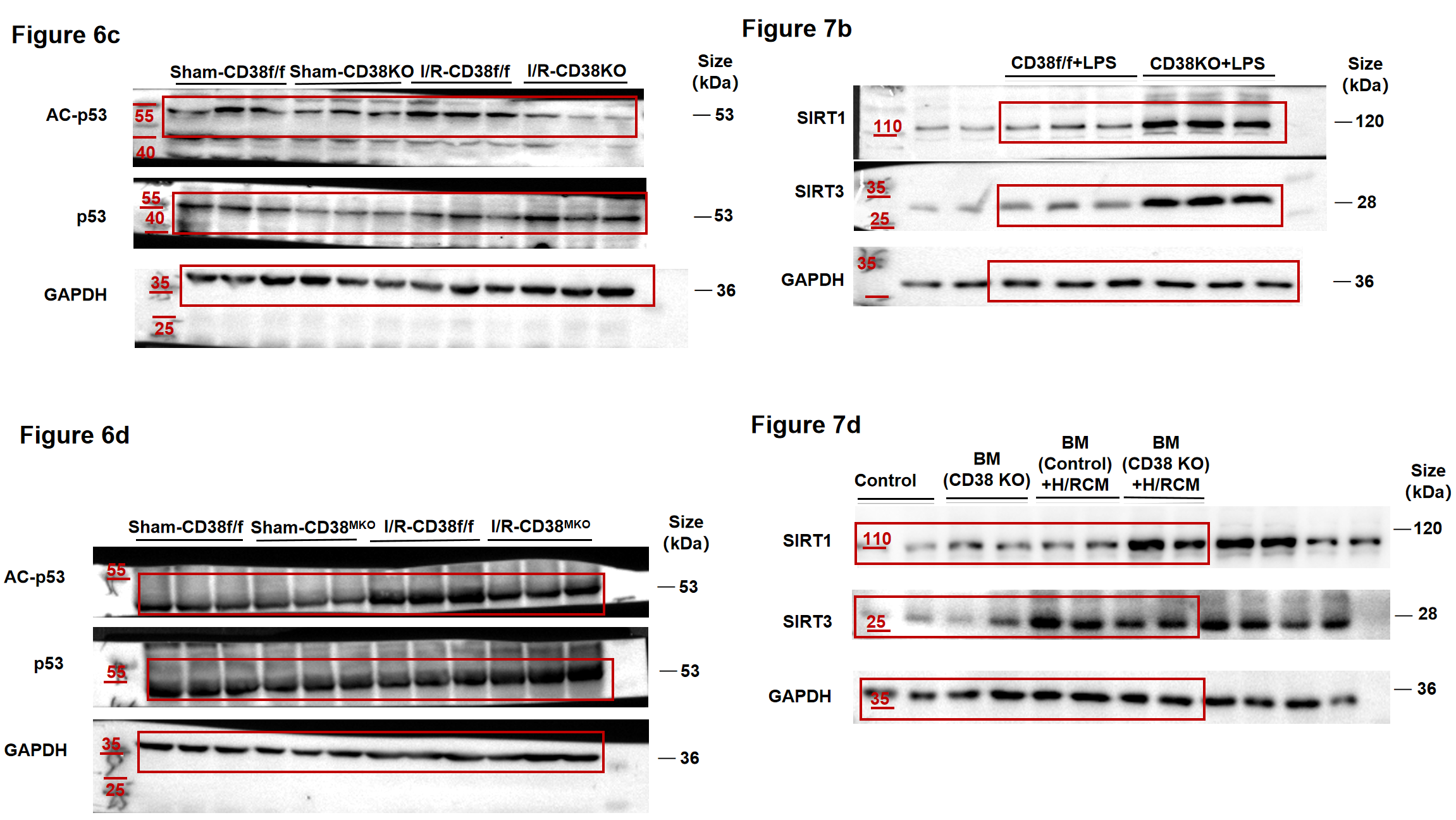


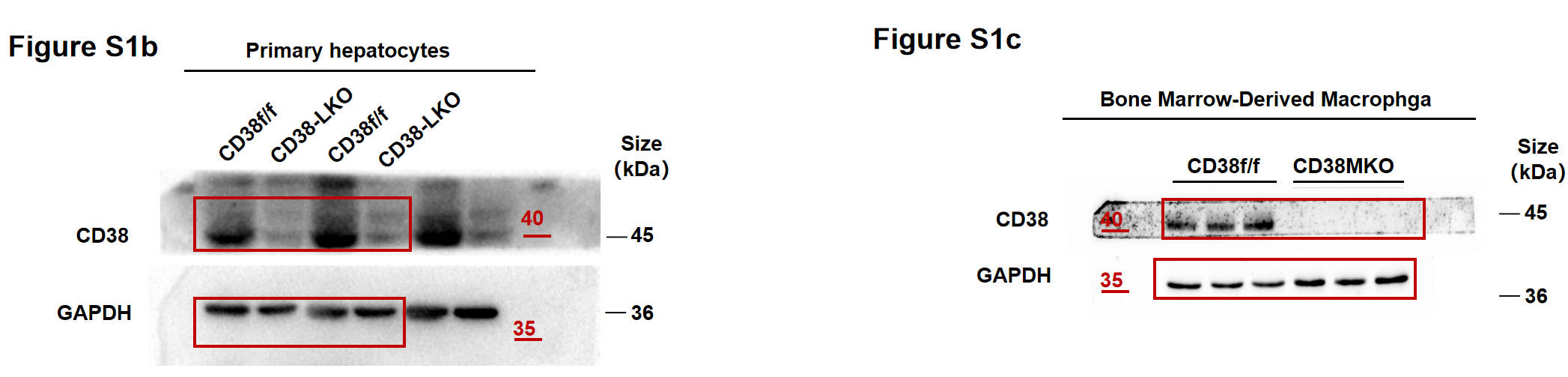

Supplement: Supplementary file 1 — Supplementary data [file 41392_2025_2233_MOESM1_ESM.docx]
